# Diverse Roles of Inhibitor of Differentiation 2 in Adaptive Immunity

**DOI:** 10.1155/2011/281569

**Published:** 2011-03-17

**Authors:** Lucille Rankin, Gabrielle T. Belz

**Affiliations:** Division of Molecular Immunology, Walter and Eliza Hall Institute of Medical Research, 1G Royal Parade, Melbourne, VIC 3052, Australia

## Abstract

The helix-loop-helix (HLH) transcription factor inhibitor of DNA binding 2 (Id2) has been implicated as a regulator of hematopoiesis and embryonic development. While its role in early lymphopoiesis has been well characterized, new roles in adaptive immune responses have recently been uncovered opening exciting new directions for investigation. In the innate immune system, Id2 is required for the development of mature natural killer (NK) cells, lymphoid tissue-inducer (LTi) cells, and the recently identified interleukin (IL)-22 secreting nonconventional innate lymphocytes found in the gut. In addition, Id2 has been implicated in the development of specific dendritic cell (DC) subsets, decisions determining the formation of **αβ** and **γδ** T-cell development, NK T-cell behaviour, and in the maintenance of effector and memory CD8^+^ T cells in peripheral tissues. Here, we review the current understanding of the role of Id2 in lymphopoiesis and in the development of the adaptive immune response required for maintaining immune homeostasis and immune protection.

## 1. Introduction

Protective immunity relies on the differentiation and maturation of lymphocytes into different cell lineages and the subsequent development of effector functions. Common lymphoid progenitors (CLPs) found in the bone marrow give rise to multiple different lineages including B, T, natural killer (NK) cells, NK T (NKT) cells, dendritic cells (DCs), and lymphoid tissue-inducer (LTi) cells. In the peripheral tissues, these lineages undergo further diversification as they mature. 

Transcription factors play a key role in the commitment of lymphocytes to specific lineages. This is achieved through alteration of gene expression profiles in response to extrinsic signals (such as cytokines) that progressively restricts the developmental potential of these progenitors as they mature. Distinct transcriptional programs drive the proliferation, survival, and differentiation of lymphocytes into functionally different cell types. Dysregulation of these programs can often lead to inflammatory or autoimmune disease, cancers, and a compromised immune response. Thus, understanding how appropriate lineage-specific gene expression is achieved and maintained at steady state and in response to a pathogen challenge is important. 

The basic Helix-Loop-Helix (bHLH) group of transcription factors, the E proteins, and their inhibitors, the inhibitor of DNA binding (Id) proteins, have been shown to play distinct and fundamental roles in the regulation of lymphocyte differentiation [1, 2].

## 2. E Proteins

E proteins are a class of transcription factors that consist of HEB, E2-2, and the E2A gene products E12 and E47 that are produced by alternative splicing. They belong to a family of bHLH transcription factors that regulate gene expression of downstream targets by binding or associating with consensus E-box sequences in DNA [3]. These sequences are present in the regulatory regions of a number of lineage-specific genes such as CD4 in T cells and mannose-binding lectin 1 (Mbl1) in B cells [4]. Members of the bHLH family are defined by two conserved domains—the HLH domain regulates homo- and/or heterodimerization while their basic domain plays a part in the binding of dimers to DNA [5].

The E protein, E2A, is essential for the development of committed B lymphocyte progenitors from CLPs (Table 1) [6, 7]. E2A-deficient mice completely lack B-cells due to the requirement of E2A for pro-B-cell development into mature B cells prior to the IgH D-J rearrangement [8]. Interestingly, E2A regulates this transition in a dose-dependent manner which is reflected in an ~50% reduction in the prevalence of pro-B cells in E2A^+/−^ mice [7, 8]. However, E proteins do not function in isolation to control lymphocyte development. For example, the Early B-cell Factor (EBF)-1 and Pax5 transcription factors are also key determinants in B-cell differentiation and restrict cells at the pro-B-cell stage to adopt a B-cell fate [9, 10]. Through biochemical and genetic analysis, it has been shown that E2A acts upstream of Pax5 and EBF during pro-B-cell development [11–13]. Thus, E2A is likely to be one of the earliest transcription factors that positively directs lymphocyte progenitors into the B-cell lineage.

E proteins are also involved in the stepwise developmental steps of T cells from CD8^−^CD4^−^ double negative (DN) to CD8^+^CD4^+^ double positive (DP) to single positive CD8^+^ T cytotoxic cells or CD4^+^ T helper cells [14, 15]. These proteins have been shown to function together as heterodimers in T-cell development [15]. Thymocytes express multiple E proteins that have previously been thought to play complementary and compensatory roles during early T-cell differentiation. It has since been shown that HEB was uniquely required for the development of CD8^+^CD4^+^ double positive stage of T-cell development and for the development of invariant natural killer T cells at an early progenitor stage [16]. Only relatively mild defects in T-cell development were observed in mice lacking either E2A or HEB, but mice lacking both proteins or dominant negative HEB mutants had severe T-cell deficiencies due to a partial blockade of developing T cells at the DN stage of TCR*β* gene rearrangement [14, 17–20]. Thus, these studies demonstrate the existence of functional redundancy within the E protein family. A role of E proteins in regulating NKT cells had not been previously reported, but the E protein antagonist Id2 has been implicated in regulating NKT cell homeostasis, suggesting that such a link may exist [10].

## 3. Id Proteins

**Figure 1:**
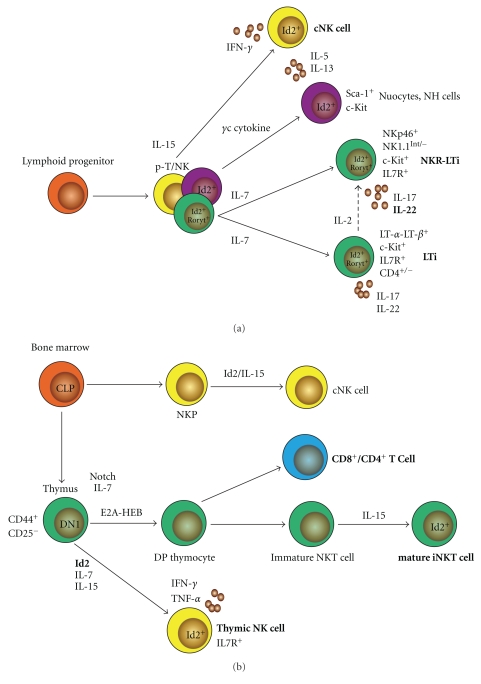
Regulation of NK, NKT, and innate lymphoid cells by Id2 during development. (a) Distinct subsets of lymphoid cells develop from hematopoietic cell precursors in the fetal liver in an Id2-dependent manner. Innate lymphoid cells can be divided into three main branches—(i) NK family, (ii) nuocytes/natural helper cells, and (iii) LTi/NKR-LTi cells that are dependent on Ror*γ*t. The NK cell lineage is characterised by its spontaneous cytotoxicity and dependence on IL-15 for development. They develop from a bipotent T/NK cell precursor (pT/NK). The second group of cells contain the nuocytes and natural helper (NH) cells that facilitate B-cell responses. Although these cells depend on IL-7 and IL-2 for their development, they do not require Ror*γ*t. The Ror*γ*t-dependent branch includes LTi cells and innate lymphoid cells (including NKR-LTi cells) which produce the cytokines IL-17 and IL-22 and require IL-7 signaling. (b) Distinct subsets of lymphoid cells develop from a common lymphoid progenitor in the bone marrow of the adult mouse and upregulate or require Id2 during development—(i) conventional NK cells (cNK), (ii) thymic NK cells, and (iii) NKT cells. NK cell progenitors upregulate Id2 and receive IL-15 signals to become mature cNK cells. Thymic NK cells develop from a CD44^+^CD25^−^ bipotent NK/T cell precursor where upregulation of Id2 leads to an NK cell fate. These cells differ from cNK cells as they require both IL-7 and IL-15 signaling for development. They also have an enhanced ability to secrete TNF-*α* and IFN-*γ* compared with cNK cells. NKT cells develop from T cell-committed double-positive (DP) thymocytes. During maturation of NKT cells in the thymus, Id2, together with an array of surface markers, is upregulated and culminates in NKT cells that are able to expand into the periphery.

The four Id proteins, Id1-4, are closely related in their HLH regions but differ in their tissue distributions [21, 22]. Id proteins dimerize with ubiquitously expressed E proteins through their highly conserved HLH motif. In contrast to E proteins, Id proteins lack the adjacent basic region necessary for DNA binding; thus, Id/E protein complexes inhibit E protein binding to DNA. Id proteins have many functions during early lymphopoiesis including B-cell specification and in directing the divergence of NK, *αβ*, and *γδ* T-cell lineages in early thymopoiesis (Figure 1, Table 1) [23–26]. Overexpression of Id1, Id2, or Id3 appears to have similar effects on lymphocyte development and block both B-cell and T-cell development [27–30]. The specific action of Id proteins in controlling lineage fate decisions depends on the ratio of Id to E proteins in developing cells. These levels can be modulated by extrinsic factors such as cytokines. In addition to E proteins, Id proteins have also been shown to interact with other transcription factors including the retinoblastoma protein (Rb), the ETS (E-twenty six), and Pax (Paired box) families to control cell differentiation [31–33].

## 4. Id2, LTi Cells, and Lymphoid Tissue Development

**Figure 2:**
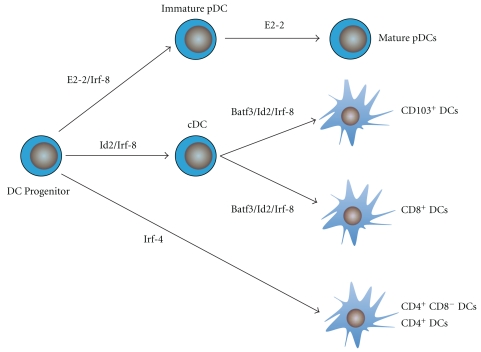
Transcription factors involved in DC development. Several transcription factors are required for the development of the common DC progenitor into functionally and phenotypically distinct subsets. IRF-8 is required for the development of both pDC and some cDC populations including the CD103^+^ and CD8*α* ^+^ DC subsets. The bHLH protein E2-2 specifies the pDC lineage and is also required for the maintenance of mature pDC identity. During differentiation, Id2 is induced in all conventional DC subsets and binds E2-2 to prevent their development into pDCs. The transcription factor Batf3 is essential for the development of CD103^+^ DCs; however, precursors of CD8*α* ^+^ DCs can develop in its absence although their survival is impaired. Other DC (CD4^+^ and CD4^−^CD8^−^ DCs) subsets depend more specifically on IRF-4 for their development.

Id2 is a critical regulator of multiple steps in development of lymphoid tissue and lymphocyte differentiation (outlined in Figures 1 and 2). Mice deficient in Id2 fail to develop lymph nodes, Peyer's patch, and other secondary lymphoid tissues including nasal-associated lymphoid tissues (NALTs) [26, 34]. This is attributable to the lack of LTi cells (Figure 2) [25, 26]. 

LTi cells are essential for the development and organization of secondary lymphoid tissue and appear to play a key role in the maintenance and restoration of these tissues after destruction from disease [34–39]. LTi cells are classically defined as lineage negative (CD3^−^CD19^−^NK1.1^−^Gr.1^−^CD11c^−^) CD45^+^c-kit^low^IL-7R^+^CD4^+/−^ [38] and are thought to originate in the fetal liver from IL-7R^+^Sca-1^low^c-kit^low^ progenitors [40]. They depend on IL-7 signalling which acts through IL-7R leading to expansion of the LTi cell pool for lymphoid tissue organogenesis [41, 42]. Unexpectedly, LTi cells have been shown to produce IL-17 and/or IL-22 which are not required for lymph node formation but are critical for tissue remodelling [43, 44]. Both Id2 and Ror*γ*t are required for induction of the signaling cascade involving IL-7R and LT*α*1*β*2/LT*β*R that eventually leads to the formation of lymph nodes and Peyer's patch. The formation of NALT, however, appears to be independent of Ror*γ*t and LT*β*R signaling [26, 34, 45]. Id2 is thought to regulate the differentiation of a common ancestor to LTi cells responsible for NALT, Peyer's patch, and lymph node organogenesis, and it is possible that Ror*γ*t is required further downstream to augment the signalling cascade. Studies of knockout and transgenic mice including retinoic acid-related orphan receptor (Ror) *γ*t^−/−^ (Ror*γ*t^−/−^), LT*α* ^−/−^, CXCR5^−/−^, IL-7R^−/−^, Janus Kinase (JAK) 3^−/−^, and Thymocyte selection-associated high-mobility group box (TOX)^−/−^ mice have shown that in addition to Id2, Peyer's patch organogenesis and/or development of functional LTi cells also depend on these molecules [36, 46–49].

## 5. Id2 and Innate Lymphoid Cells

Innate lymphoid cells have recently emerged as a novel family of diverse hematopoietic effector cells that serve protective roles in immune responses to infectious organisms, in lymphoid tissue formation, and in the homeostasis of stromal cells. Collectively, they include NK cells and LTi cells in addition to nonclassical innate lymphoid cells that can produce IL-5, IL-13, IL-17, and/or IL-22 (Figure 1). These lineages appear to be developmentally related requiring both the expression of Id2 and cytokine signalling through the common *γ*-chain of the IL-2 receptor. Functionally, these cells are quite heterogeneous (Figure 1(a)). For example, innate lymphoid cells that secrete the cytokines IL-5 and IL-13 are found in the adipose tissue associated with the mouse mesentery. These lin^−^c-kit^+^sca-1^+^ cells have been termed “nuocytes” or natural helper cells reflecting their ability to provide help to the B1 cells [50]. Another class of nonclassical innate lymphoid cells are characterized by their expression of the NK cytotoxicity receptor, NKp46 (encoded by *NCR1*, the natural cytotoxicity receptor), and their ability to produce IL-22. They have recently been described as NKR-LTi (NK receptor LTi) cells and are found predominantly in the intestinal lamina propria and Peyer's patches, as well as other mucosal compartments including the mesenteric lymph nodes [51–53]. They are dependent on the expression of Ror*γ*t, have negligible levels of expression of NK1.1, and lack cytotoxic functions [53]. Although these cells are defined by their surface expression of NKp46, they do not depend on *NKp46*/*NCR1* for their development [53]. NKR-LTi cells are more closely related to LTi cells than NK cells as both LTi and NKR-LTi lineages depend on Id2 and Ror*γ*t for their development, express c-kit, require IL-7 signaling, and secrete both IL-22 and IL-17 in response to IL-23 signaling through the IL-23R [52]. However, only a small fraction of NKR-LTi cells produce IL-17 [53, 54]. NKR-LTi cells are thought to play an important role in intestinal immunity as they significantly reduced in germ-free mice implying that signals from gut microflora drive their proliferation and survival [53, 55]. 

NK, LTi, and NKR-LTi cells depend on Id2 for their development, raising the possibility that they may stem from a common progenitor. *In vitro*, CD4^+^CD3^−^ LTi cells were shown to differentiate into NK cells when cultured in IL-2, which lead to the proposal that this population contains progenitors of NK cells [38]. Further analysis has elucidated that the different Id2-dependent lineages found in the gut - LTi, NKR-LTi, and NK cells may develop along different pathways driven by specific cytokines. IL-7 is required for the proliferation of LTi and NKR-LTi cells which are dependent on Ror*γ*t. In man, LTi and NKR-LTi cells also depend on Ror*γ*t (RORC in humans) for their development, while NK cells arise from RORC^−^lin^−^c-kit^+^KLRG1^+^IL-7R^−^ precursors [56]. It is IL-15 that drives the differentiation of NK precursors into mature NK cells *via* a Ror*γ*t-independent pathway [56]. This is supported by the finding that a severe loss of mature NK cells occurs in IL-15^−/−^ mice, while LTi and gut NKR-LTi cell numbers are similar to those in wild-type mice [53]. Further lineage relationship analysis by inducible fate mapping of Ror*γ*t and *in vitro* cultures has shown that LTi and NKR-LTi cells stem from various subsets of foetal liver precursors based on Ror*γ*t and *α*_4_ *β*_7_ expression, while NK cells arise from a Ror*γ*t^−^ *α*_4_ *β*_7_^+^progenitor [57]. Although LTi and NKR-LTi cells share some characteristics with NK cells, they are developmentally and functionally distinct from NK cells. Precisely how LTi and NKR-LTi cells are related is a question that has received much attention in recent years. *In vitro* culture and *in vivo* transfer experiments have shown potential developmental plasticity between these two cell types. In this setting, Ror*γ*t^+^ LTi-derived cells were able to upregulate NKp46 suggesting that LTi-like cells may be direct progenitors of NKR-LTi innate immune cells but not NK cells [58].

## 6. Id2 and NK Cells

NK cells play an essential role in immune surveillance and defence against intracellular pathogens. They develop from CLPs in the bone marrow but also arise in the fetal liver and thymus from a more restricted T/NK cell progenitor. Here, the most immature but committed NK cell progenitors are defined by their expression of the IL-2/IL-15-*β* receptor, CD122, and the absence of the T lymphocyte markers CD3, CD4, and CD8 [59, 60]. NK cells are dependent on the cytokine IL-15 for their proliferation and expansion into mature NK cells [61, 62]. Several transcription factors including Ets-1, MEF-1, PU.1 [63–65], and Id2 are required for the development of the CD122^+^ NK lineage cells in the thymus and bone marrow-derived mature NK cells in the spleen [25, 26, 66]. 

Although the development of T cells in the thymus is well defined, thymic NK cell development from a bipotent NK/T cell progenitor and the mechanisms regulating this pathway are less well studied. Analysis of NK/T cell progenitor activity in the fetal thymus demonstrated that the most immature thymocytes, the CD44^+^CD25^−^ stage, contain NK development potential. This potential continues until cells reach the CD44^+^CD25^+^ stage [76]. E2A-deficient mice have a partial block at the earliest stages of T-cell development in the thymus where NK/T cell progenitors arise [14, 17]. In addition, HEB^−/−^ and the transheterozygous E2A^+/−^HEB^+/−^ mice also display defects in the transition from the double-negative to the double-positive stage of T-cell maturation [15, 74, 75]. 

Id2-deficient mice display a severe reduction in thymus-derived NK cells. It appears to play differential roles in NK cell development in the bone marrow and thymus. In the bone marrow, Id2 is not thought to be required for the development of committed NK progenitor cells but instead acts to regulate the formation of mature NK cells [25]. This maturation process is at least in part regulated by interactions between Id2 and E2A activity as loss of both Id2 and E2A restores the development of mature NK cells. Despite this, these cells are impaired in their ability to emigrate from the bone marrow to spleen or in peripheral blood. Thus, overexpression of E-box proteins in this setting may contribute significantly to the lack of NK cell development in Id2-deficient mice [25].

The collaborative role of Id2, HEB, and IL-15 in NK cell development has been further investigated. It has been hypothesised that the cytokine IL-15 and Id2 work synergistically to drive differentiation of NK cells in the thymus [77]. Id2 is expressed in the thymic CD1*α* ^−^CD5^+^precursor that has both NK- and T-cell potential. These cells could be expanded and differentiated into mature NK cells in the presence of IL-15 [77]. In this setting, induction of Id2 promoted NK cell development but impaired T-cell differentiation. This pathway could be reversed by coexpression of HEB suggesting that the balance between Id2 and HEB in the early progenitor cells is an important factor in determining NK/T cell fate. Indeed overexpression of HEB leads to commitment to the T-cell lineage, while high levels of expression of Id2 commit progenitors to an IL-15 responsive NK-cell lineage [77].

## 7. Id2 and Natural Killer T Cells

Natural Killer T (NKT) cells are defined by their expression of CD4, NK1.1, and CD44. NK T cells have characteristics of both CD8^+^/CD4^+^T cells and NK cells [78]. They can be divided into three main subsets, the most studied being the invariant V*α*14 T-cell receptor expressing NKT (iNKT) cell population [79]. These cells also use several transcription factors such as Id2 and cytokines that are also key regulators of NK and T-cell differentiation (Figure 1(b)). Similar to NK cells, Id2 is highly expressed in NKT cells compared with naïve CD4^+^T cells suggesting it also plays a role in NKT cell development. Indeed, it has been found that Id2-deficient NKT cells have reduced expression of the homing receptor, CXCR6, and the proapoptotic molecule, Bim [80]. Regulation of these two molecules by Id2 and E protein transcription factors appears to be important in regulating the survival and accumulation of NKT cells in the liver [10].

## 8. Id2 and DCs

DCs are professional antigen-presenting cells that act as sentinels in the body and protect against pathogen infection. DCs originate from hemopoietic precursors and differentiate into a variety of subsets that have been defined based on their anatomical location, phenotypic appearance, and their capacity to take up, process, and present antigens. DCs are divided into two major populations—namely the conventional DCs (cDCs), which are highly efficient in taking up and presenting antigens, and plasmacytoid DCs (pDCs), that produce abundant amounts of interferon (IFN)-*α* in response to viral infection [81, 82]. 

Several transcription factors including Id2, interferon regulatory factors (IRFs)-2, 4, and 8, Stat3, Gfi-1, and PU-1 have been implicated in the development and/or homeostasis of DC populations [83–89]. Precisely where and how these transcription factors act to define DC subset specification is less clear. All DC subsets arise from Flt3R-expressing myeloid or lymphoid precursors and depend on the expression of the transcription factors PU.1 and IRF-8 [89–93]. Recently, Id and E proteins have been found to be critical for lineage specification of cDCs and pDCs (Figure 2). pDC fate is specifically determined by E2-2 (Tcf4) which also induces the expression of the transcription factors Spi-B, IRF-8, and IRF-7 required for their function [73, 94, 95]. Furthermore, ectopic expression of Id2 in multipotent progenitors prevented pDC, but not cDC, development presumably by inhibiting E2-2 function [96]. cDC lineages do not depend on E2-2 but instead rely on IRF-8, Id2, and the basic leucine zipper transcription factor, *ATF-like 3* (or *Batf3*), for subset specification [68, 84, 97]. During DC differentiation, Id2 expression is upregulated and this effect is strongly enhanced by the addition of TGF-*β*, suggesting that Id2 is crucial for the development of certain DC subsets [68]. Mice that lack Id2 fail to develop CD8*α* ^+^ DCs, LCs, and the more recently described CD103^+^ DC subset which shares many features with CD8*α* ^+^ DCs [68]. Complementing these findings, mice deficient in TGF-*β* lack LCs suggesting a link between TGF-*β* induction and Id2. Intriguingly, mice that lack the transcription factors IRF-8, Id2, or Batf3 present with similar defects—that is, they have significantly reduced numbers of CD8*α* ^+^ and CD103^+^ DCs. This initially suggests that these DC subsets may be highly related. The exact action of each of these transcription factors in regulating the steps involved in DC subset differentiation is not yet defined. It is also not known how different E proteins are induced and regulated in DC subsets although the balance between Id2 and E proteins is likely to be important in subset specification.

## 9. Id2 and Peripheral T-Cell Differentiation

CD8^+^ and CD4^+^ T cells are crucial for clearance of infection by viruses, intracellular bacteria, and protozoan parasites. On recognition of foreign pathogens, naïve T cells quickly become activated and develop effector functions that enable them to eliminate these pathogens. The initial phase of expansion and acquisition of effector function is followed by a contraction phase where the majority of the reactive T cells undergo programmed cell death leaving behind a small, but relatively stable, population of memory cells. These cells are poised to quickly respond to a second encounter with the same pathogen. This differentiation process is tightly regulated by a number of transcription factors. T-bet, eomesodermin (eomes), B lymphoycyte-induced maturation protein-1 (Blimp1; also called PRDI-BF1 in humans and encoded by the *Prdm1* gene), Id2, and Bcl-6 appear to be important for the generation and maintenance of CD8^+^ T cell memory [69, 98–100], reviewed in [101, 102]. In CD4^+^ T cells, T-bet, GATA-3, IRF-4, ROR*γ*t, and Foxp3 regulate differentiation of the different lineages (reviewed in [103]). 

Id2 has recently been found to play a role in the maintenance of effector and memory CD8^+^ T cells despite the fact that Id2^−/−^ mice appear to have normal development of their T-cell compartment [26, 69]. Id2 expression is low in naïve CD8^+^ T cells, but it is markedly upregulated in antigen-specific T cells following infection *in vivo*. Mice deficient in Id2 appear to lack effector memory, but not central memory, CD8^+^ T cells, but the mechanism by which this arises is unclear. However, when Id2^−/−^ mice are infected with *Listeria monocytogenes*, the CD8^+^ T-cell response is strongly impaired even though the antigen-specific T cells appear to proliferate normally during the immune response [69]. It is known that Id proteins contribute to the regulation of cell cycle progression. Thus, it has been proposed that lack of Id2 in T cells leads to enhanced apoptosis by increased expression of Bim and CTLA-4 and reduced expression of Bcl-2 [69]. Whether apoptotic proteins are directly regulated by Id2 during differentiation or, alternately, whether cell death is the outcome of cell cycle arrest that occurs in the absence of Id2 (perhaps a consequence of disregulated bHLH partners) is still unclear. 

The regulation of CD4^+^ T-cell differentiation into effector and memory populations by Id2 has not been extensively investigated. Id2-deficient mice exhibit features of a Th2 bias with increased IgG1 and IgE and enhanced expression of Th2-related genes such as IL-4. However, this phenotype does not appear to be an intrinsic feature of Id2 deficiency in CD4^+^T cells as it does not persist in T cells *in vitro* [104]. One possible explanation for the dysregulation of Th1/Th2 balance in the Id2^−/−^ mice is that the reduced number of CD8*α* ^+^ DCs and IL-12 associated with this DC type in these mice may limit the generation of Th1 type responses [104]. Further investigations will be necessary to elucidate the specific actions of Id2 in CD8^+^ and CD4^+^ T cells.

## 10. Id2 Effects outside the Immune System

In addition to the pronounced effects Id2 has on the development of the immune system, it also plays diverse roles in other cell types pointing to the importance of this transcription factor in development. For example, Id2 has been implicated in red blood cell development through its interactions with the transcription factor PU.1 and repression of Rb protein [24, 32, 105]. Outside the hematopoietic system, Id2 is critical for the development of mammary glands. Id2-deficient female mice show lactation defects associated with impaired lobuloalveolar development, while males have significantly reduced spermatogenesis [106, 107]. Similarly, Id2-induced cell cycle arrest prevents differentiation of enterocyte precursors during embryogenesis, and Id2 expression in distal tip lung epithelial multipotent precursors is essential for normal lung formation and remodelling [108].

## 11. Conclusions and Future Directions

In the past 10 years, major steps forward have been made in understanding how Id and E proteins work to regulate lymphopoiesis. It has long been known that the generation of adaptive immunity requires a diverse set of lymphocytes with both distinct and overlapping functions. Id2 plays an important role in defining the differentiation fate of peripheral lymphocytes and in their responses to infection. Despite this progress, several important questions defining the role of Id2 in lymphopoiesis remain unanswered. Although a number of immune cell lineages require Id2 for differentiation, it is unclear precisely where Id2 acts to define fate decisions. Furthermore, how extrinsic signals such as cytokines regulate Id2 and the balance of E proteins has not been investigated. It is clear that Id2 is involved in the development of DCs, NK, LTi, and CD3^−^NKp46^+^ cells. However, it remains to be elucidated how these Id2-dependent lineages are developmentally related and how Id2 regulates their differentiation at a molecular level. For example, which cytokines induce Id2 expression and what are the downstream targets that control lineage fate decisions? Furthermore, how Id2 regulates the balance between effector/memory T-cell generation and differentiation plasticity is poorly characterized. It is likely that detailed elucidation of the functions of Id2 and its regulation will lead to a deeper understanding of how this transcription factor contributes to the overall process of immune homeostasis and protective immunity.

## Figures and Tables

**Table 1:** Phenotype of mouse strains lacking Id and E proteins.

Gene	Phenotype	Reference
Id1	No significant phenotype in Id1^−/−^ Id1^−/−^/E2A^−/−^ knockout mice has improved postnatal survival compared to E2A^−/−^ mice	[67]
Id2	Lack lymph nodes, Peyer's patch and NALT Effects in memory CD8^+^ T cell maintenance Lack NK cells Lack CD8*α* ^+^ DCs and Langerhans cells	[26, 34, 68, 69]
Id3	Overexpression promotes NK cell development Defects in B and T cells Increase in *γδ* T cell production	[70] [71]
Id4	Smaller brain size Block in differentiation of neural progenitors Role in lymphopoiesis not yet investigated	[72]
E2A	Increased number of NK cells Ablated B-cell development	[6]
E2-2	Lack plasmacytoid DCs	[73]
HEB	Disruption of *αβ* thymopoiesis from the DN to DP stage	[15, 74, 75]

## Abbreviations

Blimp1: B lymphocyte-induced maturation protein-1
CLP: Common lymphoid precursor
DCs: Dendritic cell(s)
cDCs: Conventional dendritic cell(s)
cNK: Conventional NK cell(s)
pDCs: Plasmacytoid dendritic cell(s)
FLT3R: Fms-like tyrosine kinase 3 receptor
GALT: Gut-associated lymphoid tissues
HLH: Helix-loop-helix; bHLH, basic helix-loop-helix
Id: Inhibitor of DNA binding
IFN: Interferon regulatory factor
LTi: Lymphoid tissue inducer cell(s)
NALT: Nasal-associated lymphoid tissue
NK: Natural killer cell(s)
NKR-LTi: NK receptor LTi cell(s)
NKT: NK T cell(s)
Rb: Retinoblastoma
Ror*γ*t: Retinoic acid-related orphan receptor *γ*t.
